# Dysregulated Immune and Metabolic Microenvironment Is Associated with the Post-Operative Relapse in Stage I Non-Small Cell Lung Cancer

**DOI:** 10.3390/cancers14133061

**Published:** 2022-06-22

**Authors:** Shirong Zhang, Xiao Xiao, Xiuli Zhu, Xueqin Chen, Xiaochen Zhang, Jingjing Xiang, Rujun Xu, Zhuo Shao, Jing Bai, Yanping Xun, Yanping Jiang, Zhengzheng Chen, Xuefeng Xia, Hong Jiang, Shenglin Ma

**Affiliations:** 1 Key Laboratory of Clinical Cancer Pharmacology and Toxicology Research of Zhejiang Province, Cancer Center, Affiliated Hangzhou First People’s Hospital, Zhejiang University School of Medicine, Hangzhou 310006, China; shirleyz4444@zju.edu.cn (S.Z.); xiangcell@163.com (J.X.); xu_rj@hotmail.com (R.X.); 15988855262@126.com (Y.X.); kio6899@163.com (Y.J.); 2 Translational Medicine Research Center, Affiliated Hangzhou First People’s Hospital, Zhejiang University School of Medicine, Hangzhou 310006, China; 3 Geneplus-Shenzhen, Pingshan District, Shenzhen 518118, China; xiaoxiao@geneplus.org.cn; 4 Geneplus-Beijing, Changping District, Beijing 102206, China; zhuxl@geneplus.org.cn (X.Z.); shaozhuo@geneplus.org.cn (Z.S.); baijing@geneplus.org.cn (J.B.); xuefengx@gmail.com (X.X.); 5 Department of Medical Oncology, Affiliated Hangzhou Cancer Hospital, Zhejiang University School of Medicine, Hangzhou 310005, China; chenlucy1437@aliyun.com; 6 Department of Medical Oncology, The First Affiliated Hospital, Zhejiang University School of Medicine, Hangzhou 310003, China; zhangxiaochen74@163.com; 7 Department of Pathology, Affiliated Hangzhou First People’s Hospital, Zhejiang University School of Medicine, Hangzhou 310006, China; 8 Tongxiang First People’s Hospital, Tongxiang 314500, China; txyyjykczz@163.com; 9 Department of Thoracic Surgery, Affiliated Hangzhou First People’s Hospital, Zhejiang University School of Medicine, Hangzhou 310006, China

**Keywords:** stage I NSCLC, relapse, immune and metabolic microenvironment, TATs, relapse risk prediction

## Abstract

**Simple Summary:**

The underlying mechanism of post-operative relapse of non-small cell lung cancer (NSCLC) remained poorly understood. This study highlights that both tumors and adjacent tissues from stage I NSCLC with relapse show a dysregulated immune and metabolic environment. Immune response shifts from an active state in primary tumors to a suppressive state in recurrent tumors. A model based on the enriched biological features in the primary tumors with relapse could effectively predict recurrence for stage I NSCLC. These results provide insights into the underpinning of the post-operative relapse and suggest that identifying NSCLC patients with a high risk of relapse could help the clinical decision of applying appropriate therapeutic interventions.

**Abstract:**

The underlying mechanism of post-operative relapse of non-small cell lung cancer (NSCLC) remains poorly understood. We enrolled 57 stage I NSCLC patients with or without relapse and performed whole-exome sequencing (WES) and RNA sequencing (RNA-seq) on available primary and recurrent tumors, as well as on matched tumor-adjacent tissues (TATs). The WES analysis revealed that primary tumors from patients with relapse were enriched with *USH2A* mutation and 2q31.1 amplification. RNA-seq data showed that the relapse risk was associated with aberrant immune response and metabolism in the microenvironment of primary lesions. TATs from the patients with relapse showed an immunosuppression state. Moreover, recurrent lesions exhibited downregulated immune response compared with their paired primary tumors. Genomic and transcriptomic features were further subjected to build a prediction model classifying patients into groups with different relapse risks. We show that the recurrence risk of stage I NSCLC could be ascribed to the altered immune and metabolic microenvironment. TATs might be affected by cancer cells and facilitate the invasion of tumors. The immune microenvironment in the recurrent lesions is suppressed. Patients with a high risk of relapse need active post-operative intervention.

## 1. Introduction

Lung cancer represents the peaks of both incidence and mortality among all cancer types [1]. Non-small cell lung cancer (NSCLC) is the most common histological type of lung cancer, especially in nonsmoking patients. Complete surgical resection with mediastinal lymph node dissection is the curative treatment for patients with early stage lung cancers [2]. However, due to the high relapse rates about 30–55% for those who could receive curative resection [3], five-year survival rates for fully resected stage I NSCLC still range from 50% to 70% [4].

Defining the molecular mechanism underlying recurrence is critical for identifying biomarkers which could serve as prognostic indicators or therapeutic targets to improve clinical outcomes. Multiple studies have attempted to decipher the disparity between paired primary and metastasis lesions and suggested that tumor microenvironment (TME) with immune suppression might be the mechanism leading to metastasis [5,6,7]. Although the defect of immune response is quite prominent in metastasis compared with primary tumors, TME alteration might occur early in the primary tumor and therefore facilitate the dissemination of tumor cells.

Prognostic models capable of predicting clinical outcome in early stage NSCLC have attracted much attention. A previous study utilized immune signature to predict the overall survival in early stage NSCLC [8]. Similarly, hypoxia-related signature was also proved to be effective in predicting overall survival in both early and advanced-stage NSCLC [9,10]. Apparently, the choice of signature was guided by the molecular mechanism associated with the malignant features of tumor cells. We reason that an in-depth analysis of multi-omics data [11,12,13,14] of patients with relapse could provide more valuable markers for predicting recurrence, and this might benefit a subpopulation of patients by providing evidence supporting applying certain therapeutic regimens.

Herein, we collected samples from the primary stage I NSCLC with different risks of relapse representing the early stage of their kind and nine recurrent tumors corresponding to the advanced stage. We performed whole-exome sequencing (WES) and RNA sequencing (RNA-seq) on these samples and compared the genetic and transcriptional profile between cohorts with and without relapse. We found that primary tumors from patients with post-operative relapse were marked with aberrant immune and metabolic microenvironment, and their matched tumor-adjacent tissues (TATs) had decreased immune response. By analyzing paired primary and recurrent lesions, we identified a suppressive immune status of recurrent tumors that contrasted the elevated immune response in the primary tumors of patients with recurrence. Furthermore, we established a predictive model to stratify patients into groups with different risks of recurrence.

## 2. Materials and Methods

### 2.1. Patients

We prospectively enrolled 57 patients with stage I NSCLC from 2012 to 2018, including 29 patients without relapse and 28 patients who relapsed within 5 years. Written informed consent was obtained from all participants. The paired TATs for each patient were collected as a negative control. Nine recurrent tumors were collected for WES and RNA-seq analysis. These patients underwent surgical resection and received no adjuvant therapy. All samples were collected in Affiliated Hangzhou First People’s Hospital, Zhejiang University School of Medicine. Detailed clinical characteristics are provided in Tables S1–S3. This study was conducted in accordance with the Declaration of Helsinki and approved by the Institutional Review Board of Affiliated Hangzhou First People’s Hospital, Zhejiang University School of Medicine (No. IIT-20210922-0037-01).

### 2.2. DNA Extraction and Sequencing

DNA was extracted from fresh tumor and paracancerous tissues by using QIAamp DNA MiniKit (Qiagen, Hilden, Germany) according to the manufacturer’s instructions. The concentrations of DNA were determined by Qubit fluorometer (Invitrogen, Carlsbad, CA, USA). A total of 1 µg DNA was fragmented into 200–250 bp segments, using a Covaris S2 instrument (Woburn, MA, USA). Sequencing libraries were prepared by using the KAPA DNA Library Preparation Kit (Kapa Biosystems, Boston, MA, USA) according to the manufacturer’s protocol. In brief, the end of the fragments was repaired before “A” addition, adapter ligation, amplification, and hybridization to the SeqCap EZ library. The captured DNA was recovered by using Streptavidin Dynabeads (Thermo Fisher Scientific, Waltham, MA, USA) and then amplified by PCR. Sequencing was performed by using the Geneplus-2000 sequencing platform (Geneplus, Beijing, China).

### 2.3. WES Analysis

BWA (version 0.7.12-r1039, Heng Li and Richard Durbin, Cambridge, UK) was employed to align the clean reads to the reference human genome (hg19). Picard (version 1.98, Broad Institute, Cambridge, USA) was used to mark PCR duplicates. Realignment and recalibration were performed by using GATK (version 3.4-46-gbc02625, Broad Institute, Cambridge, MA, USA). Single nucleotide variants (SNVs) were called by using MuTect (version 1.1.4, Broad Institute, Cambridge, MA, USA). Small insertions and deletions (Indels) were called by GATK. Somatic copy-number alterations were identified with CONTRA (v2.0.8, Jason Li et al., Melbourne, Australia). Mutations were considered as candidate somatic mutations only when (i) the mutation was detected in at least 5 high-quality reads, (ii) the mutation with a variant allele frequency >0.01, (iii) the mutation was not present in >1% of the population in the 1000 Genomes Project (version phase 3) or dbSNP databases (The Single Nucleotide Polymorphism Database, version dbSNP 137), and (iv) the mutation was absent from a local database of normal samples. The detailed quality-control data are provided in Table S4.

### 2.4. Identification of Enrichment of Mutated Genes in the PR Cohort

**Figure 1:**
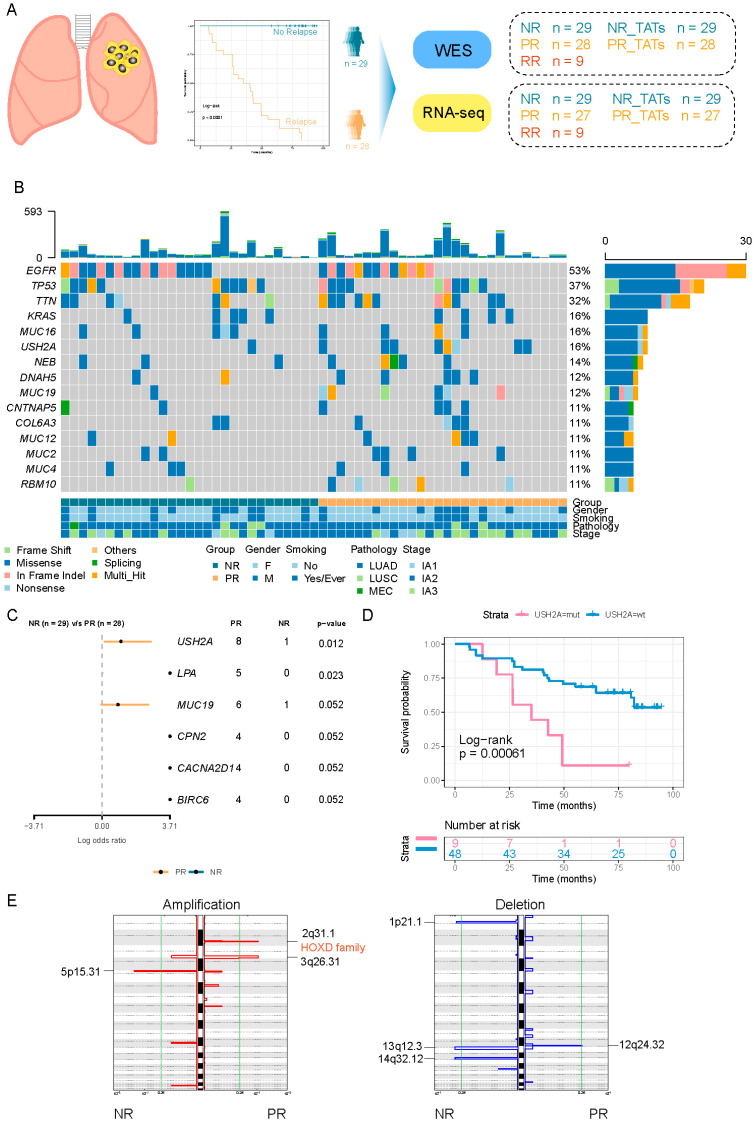
The difference of genetic characteristics between PR and NR. (**A**) Schematic diagram of the study design. (**B**) Oncoprint showing the mutations of the top 15 most frequently mutated genes. The upper barplot shows the mutation burden of each patient. The bottom annotation shows the clinical features (tumor stage, smoking, and gender). (**C**) Forest plot comparing the frequency of genes mutated in PR versus that mutated in NR. Enrichment for each gene was determined by using a two-tailed Fisher’s exact test. Only genes with a *p*-value < 0.1 are shown. (**D**) Kaplan–Meier curve for DFS of *USH2A* mutant and *USH2A* wild-type group. Log-rank test was used to compare the difference. (**E**) GISTIC 2.0 was used to detect significantly altered region with amplification (red) and deletion (blue).

The enrichment of mutant genes in the PR cohort was estimated by the odds ratio (OR) and *p*-value derived from the Fisher’s exact test. OR = prevalence in PR/prevalence in NR. Genes with OR > 1 and *p* < 0.1 are plotted in Figure 1C.

### 2.5. Signature Analysis

The R package deconstructSigs was used to analyze the single nucleotide substitution signature [15].

### 2.6. CNV Analysis

Gistic 2.0 was adopted to detect significantly recurrent CNV regions in the cohort. To evaluate the CNV level at the chromosome and arm level, we performed the following calculations as per a previous report [16]. Briefly, CNV events were identified by the log2 ratio depth of tumor to normal called from GATK (cutoff, 0.2). The lengths of aberrant regions were summed up according to the specific status (amplification or deletion), respectively. An arm-level amplification or deletion was identified when the summed-up length exceeded 50% of the arm length. When both arms of a chromosome presented CNV of the same aberrant status, a chromosome-level CNV was defined. The events of arm-level and chromosome-level were counted, respectively, generating the Arm_score and Chrom_score to represent the CNV burden [16].

### 2.7. RNA Extraction and Sequencing

RNA was extracted from fresh tissues by using TRIzol and RNeasy MinElute Cleanup Kit (Invitrogen). RNA purity was measured by using the kaiaoK5500^®^ Spectrophotometer (Kaiao, Beijing, China). RNA integrity and concentration were evaluated by using the RNA Nano 6000 Assay Kit of the Bioanalyzer 2100 system (Agilent Technologies, Palo Alto, CA, USA). According to the manufacturer’s protocol, mRNA libraries were prepared by using the NEB Next^®^ Ultra™ RNA Library Prep Kit (NEB, Beverly, MA, USA). The constructed RNA-seq libraries were sequenced on the Geneplus-2000 sequencing platform (Geneplus, Beijing, China).

### 2.8. Gene Expression Profiling and Data Analysis

The sequencing reads containing adaptor sequences and low-quality reads were removed to obtain high-quality paired-end reads. Reads passing quality control were aligned to the human genome (hg19), using HISAT (v2.0.4, Daehwan Kim et al., Baltimore, USA). Transcript assembly was performed by using StringTie (v1.2.3, Mihaela Pertea et al., Baltimore, USA). The detailed quality-control data are provided in Table S5. R package limma was used for gene differential expression analysis. Gene set enrichment analysis (GSEA) was used to calculate the enrichment scores of certain pathways. The normalized enrichment score (NES) was obtained for each patient. Gene sets with FDR values < 0.25 and NES > 0 were considered as significantly enriched.

### 2.9. Analysis of Immune Cell Infiltration

GSVA package was implemented to perform single sample gene set enrichment analysis (ssGSEA) related to immune cells [17]. The fractions of 22 types of immune cells associated with innate and adaptive immunity were derived by using CIBERSORT as previously described [18].

### 2.10. Inference of Clonal Evolution

The subclones in each sample were inferred by PyClone based on cellular prevalence and copy number of mutations [19]. The phylogeny of subclones was constructed by CITUP (github.com/sfu-compbio/citup, accessed on 20 November 2021) and further visualized by Timescape (www.bioconductor.org/packages/release/bioc/html/timescape.html, accessed on 20 November 2021).

### 2.11. Survival Analysis

A Kaplan–Meier plot was used to evaluate the difference of disease-free survival (DFS) between different groups, and the significance was assessed by a log-rank test.

### 2.12. Prediction Model Construction

WES data and RNA-seq data were used to construct recurrence prediction models independently or collectively. To calculate the mutation risk score, frequently mutated genes (prevalence ≥10%) were analyzed by univariate Cox proportional-hazards regression to evaluate their association with DFS. The two genes (*USH2A*, *RBM10*) with significant results (*p* < 0.05) were subjected to the multivariate Cox model. To calculate the expression risk score, DEGs between the PR and NR groups were analyzed by univariate Cox proportional-hazards regression to evaluate their association with DFS. Those with significant results were further selected according to their biological functions. Specifically, KEGG pathways enriched in the PR group (GSEA, NES > 2) compared with the NR group, including antigen processing and presentation, B-cell-receptor signaling, natural-killer-cell-mediated cytotoxicity, and oxidative phosphorylation, were used as the selection criteria. The selected genes were further evaluated by multivariate Cox model and Akaike information criterion. Finally, *CALR*, *HSPA2*, *PSME1*, *BRAF*, *SERPINA1*, *HLA-B*, *LTA*, and *CD48* were selected and fed into the multivariate Cox model. The univariate Cox proportional-hazards regression model was also used to evaluate the significance of clinical parameters. The risk score for the above Cox models were calculated by using the function “predict” from the stats package. Receiver operating characteristic (ROC) analysis of the model and the areas under the curve (AUC) was calculated to reflect the prediction power. The difference of DFS between high-risk and low-risk groups was determined by using a Kaplan–Meier curve and log-rank test. The optimal cutoff value was determined by the function “surv_cutpoint” from survminer package, which determines the optimal cutpoint for one or multiple continuous variables by using the maximally selected rank statistics from the maxstat R package. This is an outcome-oriented method providing a value of a cutpoint that corresponds to the most significant relation with an outcome.

### 2.13. Statistical Analysis and Visualization

Statistical analysis and plot generation were performed with R software (v 4.0.2) and GraphPad Prism (v 8.0.2), as appropriate. In all analyses, comparisons between two groups were based on two-sided Wilcoxson sum-rank test. The *p*-values < 0.05 were considered statistically significant, except for specific indications.

## 3. Results

### 3.1. Clinical Characteristics of Patients

We collected 57 primary tumor samples from stage I NSCLC patients (one sample from each patient) during surgical resection, among whom 28 patients manifested with relapse within 5-year follow-up, and their primary tumors were termed as PR in the following description, while the remaining 29 patients presented no relapse, and tumors derived from them were termed as NR. Nine patients underwent a second surgery after relapse and the resected tumors (recurrent lesion from patients with relapse, RR) were obtained. TATs matched with NR and PR were termed as NR_TATs and PR_TATs, respectively (Figure 1A). The summary of clinical characteristics is listed in Table S1.

### 3.2. The Genetic Characteristics of PR and NR

To investigate the genetic features in stage I NSCLC with a different risk of recurrence, we performed WES on NR and PR samples (Figure 1A). We found that *EGFR* and *TP53* ranked high among the frequently mutated genes, and their prevalence was comparable between PR and NR (Figure 1B and Figure S1). An increased prevalence of several mutant genes was observed in PR compared with that in NR (Figure 1C). Among them, the *USH2A* mutation was remarkably enriched in PR (29% vs. 3%, *p* = 0.012), indicating that it might be a signature for relapse in stage I NSCLC (Figure 1C and Figure S1). We compared the DFS between *USH2A*-mutant and *USH2A*-wild-type patients and found that the *USH2A* mutation was associated with worse DFS in our cohort. However, the *USH2A* mutation was not associated with DFS in the TCGA cohort whether LUAD and LUSC were inspected, respectively, or combined as a whole (Figure S2). We also observed that the *USH2A* mutation was associated with an increased tumor mutation burden (TMB) and tumor neoantigen burden (TNB) in our cohort (Figure S3), indicating that mutation in *USH2A* might be associated with an altered immune environment [20]. We did not identify any difference of TMB and TNB between the NR and PR groups (Figure S4).

The single base substitution signature analysis demonstrated that the overall patterns between NR and PR were similar. Of note, a small fraction of Signature 11 exhibiting a mutational pattern resembling that of alkylating agents was present in NR (Figure 1C), although our patients underwent no treatment before surgery. Signature 3 associated with failure of DNA double-strand break repair by homologous recombination, and an inconspicuous fraction of Signature 21 related to microsatellite unstable tumors only manifested in the PR group (Figure S5).

Taking the copy number variation (CNV) into account, we observed that there was no significant difference in the arm-level and chromosome-level CNV between NR and PR (Figure S6). However, at the focal level, significant alterations that were unique to each group were observed (Figure 1E). Notably, the 2q31.1 segment harboring HOXD family genes was significantly amplified in the PR group (Figure 1E, left panel). As reported previously, the HOX-Centric network might confer risk to serous ovarian cancer [21,22]. Overall, despite the general similar characteristics between PR and NR, PR had several unique genetic alterations.

### 3.3. PR Harbored Aberrant Immune and Metabolic Microenvironment

**Figure 2:**
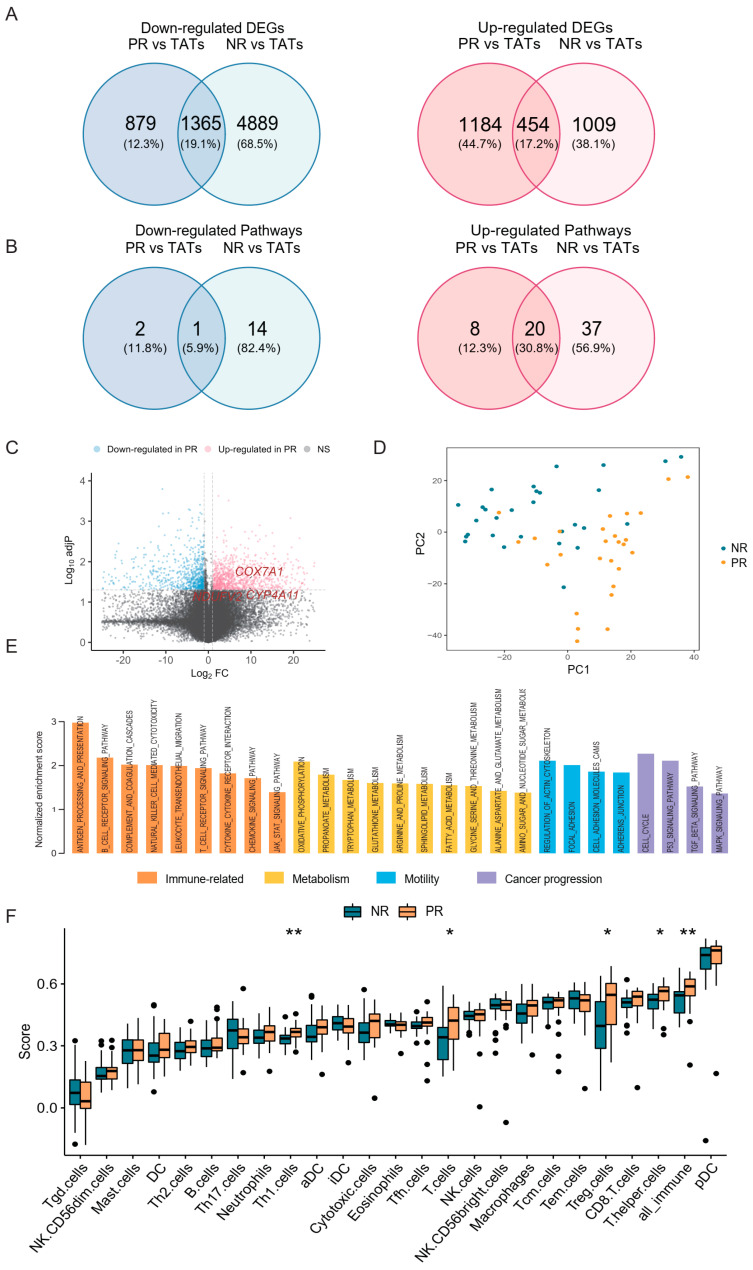
The difference of transcriptional profile between PR and NR. (**A**) Venn diagram showing the overlap of DEGs in PR and NR. DEGs, differentially expressed genes; TATs, tumor adjacent tissues. (**B**) Venn diagram showing the overlap of enriched pathways (based on DEGs) in PR and NR (see also Figure S7). (**C**) Volcano plot showing the upregulated (red) and downregulated genes (blue) in PR compared with NR. Vertical and horizontal dashed lines represent the cutoff for Log2FC (1 for upregulated and -1 for downregulated) and adjusted *p*-value (0.05). DEGs related to oxidative phosphorylation and fatty acid metabolism are indicated in red. (**D**) Principal component analysis showing the separation of NR and PR tumors. (**E**) Barplot showing the enrichment score of significantly enriched KEGG pathways in PR versus NR analyzed by GSEA. (**F**) Immune cell infiltration calculated by ssGSEA in NR and PR tumors. * *p* < 0.05, ** *p* < 0.01.

To further investigate the transcriptional profile of stage I NSCLC patients with different relapse risks, we analyzed the RNA profile in NR and PR, as well as in the matched TATs (Figure 1A). Of the differentially expressed genes (DEGs), 1463 upregulated and 6254 downregulated genes were identified in NR compared with NR_TATs; 1638 upregulated and 2244 downregulated genes were identified in PR compared with PR_TATs. Comparing DEGs in PR (vs PR_TATs) and those in NR (vs NR_TATs), we found that most DEGs were unique to PR or NR (Figure 2A), thus indicating that these two groups harbored a different transcriptional profile. Kyoto Encyclopedia of Genes and Genomes (KEGG) pathway enrichment was performed to annotate the biological functions of DEGs, and enriched pathways were mostly upregulated in both NR and PR (Figure S7). The infiltration of lymphocytes was evidenced by the enrichment of immune-related pathways in both groups. Several cancer-related pathways, including Wnt, ErbB, and Hippo signaling pathways, were downregulated in NR tumors. Nevertheless, consistent with the findings in DEGs, PR and NR shared a small fraction of enriched pathways regarding downregulated and upregulated pathways separately (Figure 2B), thus further indicating that the transcriptional difference between the two groups from our cohort might provide underpinning of relapse risk. We next attempted to identify DEGs between NR tumors and PR tumors to extract the finer dichotomous transcriptomic programming determining the relapse potential. In total, 990 upregulated and 811 downregulated genes were found (Figure 2C). Although the first two principal components derived from the DEGs revealed a separation of NR and PR groups (Figure 2D), we did not identify significant pathway enrichment by using these DEGs. Therefore, we performed GSEA, which could consider the global pattern of gene expression in the two groups. Compared with NR, the PR group was mainly enriched with pathways related to immunity, metabolism, cell motility, and cancer progression (Figure 2E). Herein, the increased activity of several immune-related pathways in the TME of PR was noticeable, thus suggesting the early immune aberration in primary tumors with a higher risk of relapse after surgery. The metabolism activity was also boosted in PR, suggesting that cells within the niche might be more energetic. Genes related to oxidative phosphorylation and fatty acid metabolism were among the upregulated genes in PR [23,24,25] (Figure 2C).

We also analyzed the infiltration of immune cells by calculating the ssGSEA score (Figure 2F). Consistent with the above findings, the proportion of total immune cell infiltration (all_immune) significantly increased in PR compared with NR, which was likely ascribed to the increase of T cells (Figure 2F). Of note, regulatory T (Treg) cells, which could play an inhibitory role in the antitumor immunity, were remarkably elevated in PR. We also analyzed the fraction of immune cell infiltration by using CIBERSORT, which consistently showed a slight increase of Treg cells in PR; however, the difference was not significant (Figure S8A). This observation raised the possibility that these cells might be dysfunctional or non-activated despite the high level of infiltration [26,27].

Given that the *USH2A* mutation was associated with an increase of TMB and TNB in our cohort, we also investigated whether this alteration was related to immune cell infiltration. Both ssGSEA and CIBERSORT results showed that Treg cells increased in tumors with *USH2A* mutation, although the analysis from CIBERSORT did not generate a statistically significant difference (Figure S8B,C). These findings further proved the potential role of *USH2A* in affecting immune surroundings.

Collectively, the above results demonstrated that PR was fundamentally different from NR regarding the transcriptional profile and harbored an imbalanced immune microenvironment which might lead to an increased risk of post-operative relapse.

### 3.4. PR_TATs Showed an Inhibitory Immune Microenvironment

**Figure 3:**
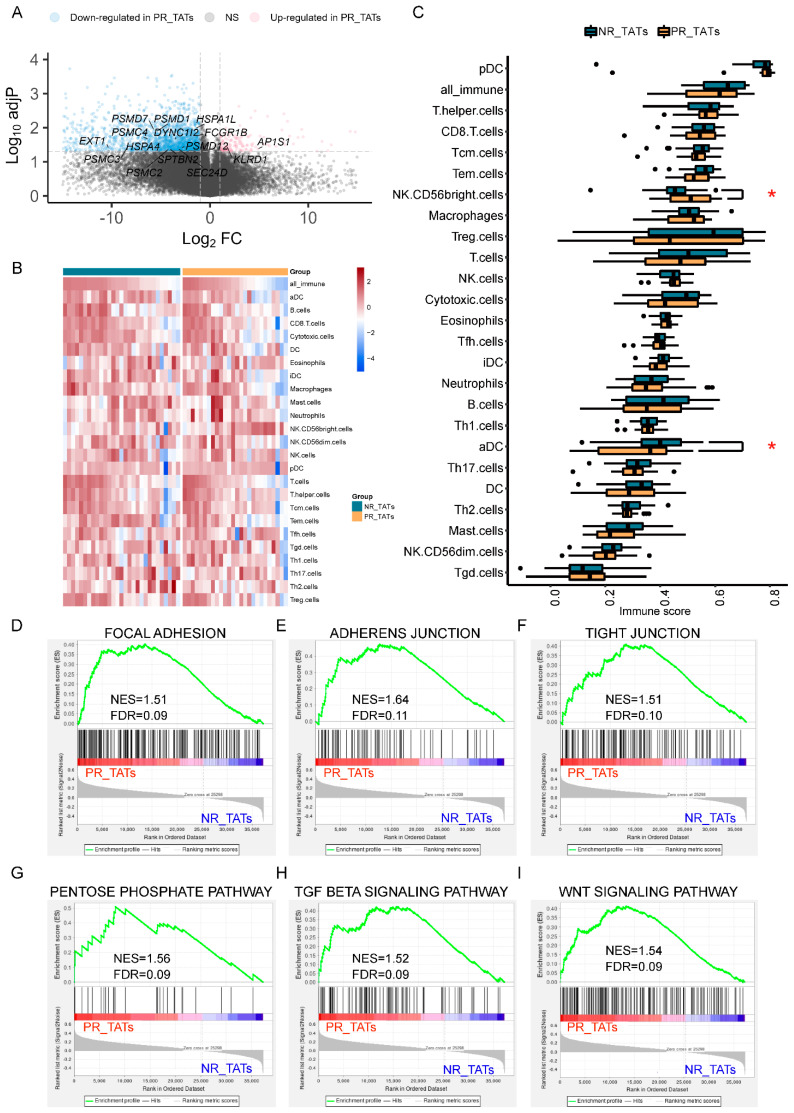
PR_TATs showed an inhibitory immune microenvironment. (**A**) Volcano plot showing the upregulated (red) and downregulated genes (blue) in PR_TATs compared with NR_TATs. Vertical and horizontal dashed lines represent the cutoff for Log2FC (1 for upregulated and -1 for downregulated) and adjusted *p*-value (0.05). Genes related to antigen processing and presentation are annotated. PR_TATs, tumor-adjacent tissues paired with PR tumors; NR_TATs, tumor-adjacent tissues paired with NR tumors. (**B**) Heatmap showing the immune cell infiltration of each sample. Samples are split into NR_TATs and PR_TATs cohorts and are in descending order according to the score of all_immune cells. For each cell type, the ssGSEA score was transformed to the scaled *z*-score before visualization. (**C**) Boxplots comparing the immune cell infiltration between NR_TATs and PR_TATs. Wilcoxon rank-sum test was performed to evaluate the statistical significance. Differences with *p* < 0.05 are indicated by red asterisks. * *p* < 0.05. (**D**–**I**) GSEA results showing the significantly upregulated pathways in PR_TATs versus NR_TATs.

Whether TATs around the tumor compartment could affect tumor growth and metastasis is unclear. We attempted to answer this question by investigating the DEGs in TATs paired with NR and PR tumors. We found that most DEGs (2174) were downregulated, while a small fraction of genes (182) was upregulated in PR_TATs compared with NR_TATs (Figure 3A). Overall, the immune state showed a suppressed tendency in PR_TATs versus NR_TATs with a group of immune-related genes falling into the downregulated genes (Figure S9) and generally decreased immune score reflecting the immune cell infiltration in PR_TATs (Figure 3B,C). Immunostimulatory aDC cells, which are a major player in antigen presentation, were lacking in PR_TATs (Figure 3C). The impaired ability of antigen processing and presentation was also manifested by the enrichment of relevant functional genes in the decreased DEGs in PR_TATs (Figure 3A). We also noticed CD56^bright^ NK cells, which were associated with compromised cytotoxicity and aggressive behaviors of tumor cells [28], were significantly enriched in PR_TATs (Figure 3C).

Furthermore, we identified several pathways that are more active in PR_TATs, using GSEA. Notably, pathways related with adhesion and junction were elevated in PR_TATs (Figure 3D–F), in addition to PR (Figure 2E), emphasizing the dual property of adhesion and junction related genes; that is, molecules involved in adhesion and junction could function in blocking the migration of cancer cells in some scenarios, as well as promoting the malignant progression of tumors under certain circumstances [29]. The pentose phosphate pathway, which was reported as a critical NAPDH origin for cancer cells to synthesize fatty acids and respond to stress [30], was also elevated in PR_TATs, indicating that this energetic alteration was not restricted to the tumors. In addition, genes involved in TGF-beta signaling and Wnt signaling were upregulated in PR_TATs, and both pathways could reshape the TME to facilitate tumor progression [31,32,33]. Taken together, although TATs were usually considered as the “normal control”, they might already undergo a series of alterations involving immune response and metabolism as a result of tumor education.

### 3.5. Genetic and Transcriptional Distinction between PR and RR

**Figure 4:**
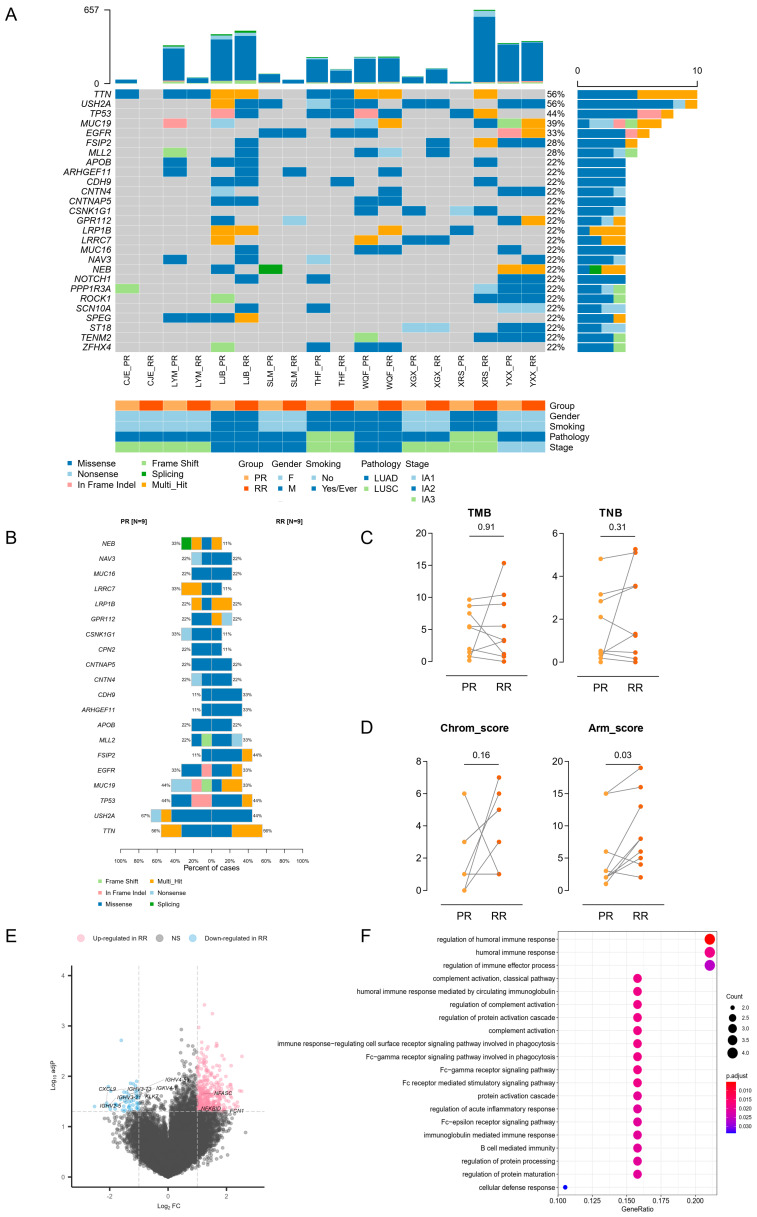
Genetic and transcriptional distinction between PR and RR. (**A**) Oncoplot depicting frequently mutated genes in paired PR and RR tumors. (**B**) Barplot comparing the prevalence of frequently mutated genes in PR and RR tumors. (**C**) Pair-wise comparison of TMB (left) and TNB (right) between PR and RR tumors. The difference was evaluated by Wilcoxon matched-pairs signed-rank test. (**D**) Pair-wise comparison of Chrom_score (left) and Arm_score (right) between PR and RR tumors. The difference was evaluated by Wilcoxon matched-pairs signed-rank test. (**E**) Volcano plot showing the upregulated (red) and downregulated genes (blue) in RR compared with PR. Vertical and horizontal dashed lines represent the cutoff for Log2FC (1 for upregulated and -1 for downregulated) and adjusted *p*-value (0.05). Genes related to immune effector process are annotated. (**F**) Dotplot showing that downregulated genes in RR versus PR enriched in immune-related Gene Ontology terms.

We also performed WES and RNA-seq in nine RR tumors (Figure 1A) and compared them with the matched PR. Pair-wise comparison demonstrated that PR and RR from most patients had a similar mutational landscape of the frequently mutated genes, except for LYM and XRS (Figure 4A). A clonal evolution analysis could help identify whether the recurrent loci was seeded from the primary lesion or formed de novo. Our results revealed that all RR samples were clonally related with their paired PR, including those from the two patients mentioned above (Figure S10). We also found that most PRs (6/9) demonstrated a low degree of intra-tumoral heterogeneity, while most RRs (7/9) had novel subclones. Given that the PR samples were acquired from stage I patients, the relatively simple clonal architecture in most cases represented an early stage of clonal evolution. Comparing the commonly altered genes between PR and RR, we found that *FSIP2* was mutated more frequently after relapse; however, the result was not significant (Figure 4B). Several studies demonstrated that *FSIP2* was associated with metastasis and drug response in breast cancers [34,35]; therefore, we suspected that *FSIP2* mutation might contribute to the survival advantage of disseminated lesions. We next compared the TMB and TNB between paired PR and RR but found no obvious difference (Figure 4C). However, we found that arm-level CNV was significantly elevated in RR, whereas chromosome-level CNV manifested no significant alteration in the pair-wise analysis (Figure 4D).

We next investigated the transcriptional profile in RR relative to PR. A small number of DEGs were identified, including 362 upregulated and 52 downregulated genes in RR (Figure 4E). Nevertheless, the downregulated compartment included several immune-related genes and were enriched for immune-related Gene Ontology terms (Figure 4E,F), suggesting an immunosuppression state of TME, which was in accordance with several previous studies [5,36,37]. We also analyzed immune cell infiltration by using ssGSEA and CIBERSORT and found generally no significant difference between PR and RR, except for decreased monocytes in RR (Figures S11 and S12).

### 3.6. Establishing A Prognostic Model for Recurrent Stage I NSCLC

In order to establish a prognostic model to predict recurrence in stage I NSCLC, we first applied univariable cox regression to analyze the association of clinical features and relapse. Age, gender, smoking status, histological subtype, tumor size, and primary site were not significantly correlated with a high risk of relapse (Figure S13A) and were therefore excluded from the model.

**Figure 5:**
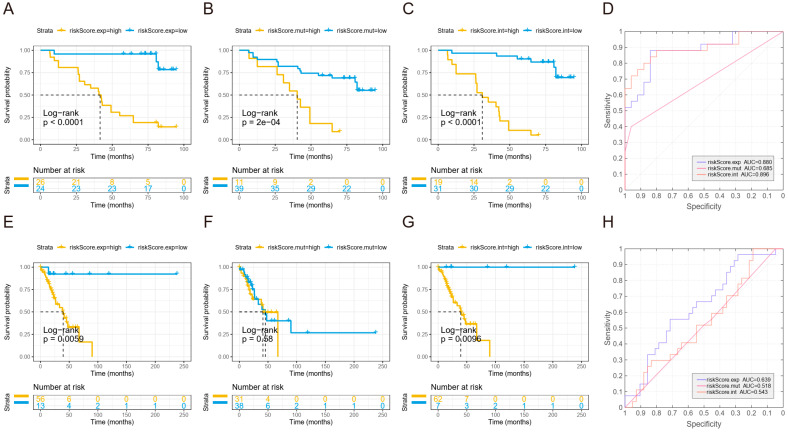
Establishing a prognostic model for predicting recurrence of stage I NSCLC. (**A**–**C**) Kaplan–Meier curve for DFS of high-risk group and low-risk groups according to expression risk score (**A**), mutation risk score (**B**), and integration risk score (**C**) calculated in the test cohort. (**D**) ROC curve for each risk score calculated in the test cohort. (**E**–**G**) Kaplan–Meier curve for DFS of high-risk group and low-risk groups according to expression risk score (**E**), mutation risk score (**F**), and integration risk score (**G**) calculated in the TCGA validation cohort. (**H**) ROC curve for each risk score calculated in the TCGA validation cohort. The cutoff values in the Kaplan–Meier plots were determined by a result-oriented function “surv_cutpoint” in survminer package.

We next sought to combine the genetic and transcriptional data to fit the model. After excluding a few non-adenocarcinoma samples to avoid bias, we restricted the training cohort to 50 adenocarcinomas. Risk scores were calculated according to the transcriptional and mutational profile, respectively (Section 2). Furthermore, an integration score was generated by combining both types of risk score. The training cohort was divided into high-risk groups and low-risk groups by the optimal cutoff value (Section 2) according to the risk score, and the high-risk groups showed inferior DFS compared to the low-risk groups whether expression risk and mutation risk were considered separately or combined (Figure 5A–C). Additionally, the mutations risk score (AUC = 0.685, hazard ratio (HR) = 1.226, 95% hazard ratio (CI) = 1.088–1.383, *p* = 0.00084) fell behind the expression score (AUC = 0.880, HR = 1.212, 95% CI = 1.068–1.177, *p* = 3.54 × 10^−6^) and integration score (AUC = 0.896, HR = 1.063, 95% CI = 1.026–1.101, *p* = 7.71 × 10^−4^) in predicting recurrence; the expression score and the integration score showed a similar performance (*p* = 0.5322, DeLong test) (Figure 5D). When we validated the model in the stage I LUAD from TCGA data, the mutation risk score could no longer distinguish high-risk patients from low-risk patients in differentiating DFS, whereas both the expression risk score and integration risk score could identify patients with a shorter DFS (Figure 5E–G). Consistently, the expression risk score (AUC = 0.639, HR = 1.557, 95% CI = 1.105–2.194, *p* = 0.0114) displayed a better performance in predicting recurrence compared with the mutation risk score (AUC = 0.518, HR = 0.9835, 95% CI = 0.8384–1.154, *p* = 0.838) and integration risk score (AUC = 0.543, HR = 0.9291, 95% CI =0.7364–1.172, *p* = 0.535) (Figure 5H). Therefore, the expression risk score generated from the transcriptional data demonstrated optimal power in predicting recurrence in both the training and validation datasets, and there is no need to integrate mutation data to boost the model. We further tested the expression risk model in another independent cohort (GSE30219) and validated that the selected genes from our model could effectively predict recurrence risk in the stage I LUAD patients (Figure S13B,C).

## 4. Discussion

Surgery remains the curative treatment for stage I NSCLC. With the expectation that surgery might completely eradicate the tumor cells, patients with clinical stage 0 or IA NSCLC after segmentectomy generally present favorable survival [38]. However, a fraction of patients will eventually relapse [39]. In the current study, we used WES and RNA-seq analysis to unveil the main distinction between patients with and without relapse and further selected genes to establish a prediction model which could effectively stratify patients from our cohorts into tiers with different risks of recurrence.

To explore the underlying mechanism associated with recurrence risk in stage I NSCLC patients, we performed systemic analysis to pin down the difference between NR and PR samples. We found that *USH2A* mutation and 2q31.1 amplification were enriched in the PR tumors. A previous study reported that the *USH2A* mutation was associated with high TMB and poor clinical prognosis. Additionally, cases with *USH2A* mutation had an increased immune score [20]. In our findings, although *USH2A* mutation tented to be positively associated with TMB and TNB, as well as the abundance of Treg cells, it was not related to the global immune infiltration, which might explain the inferior DFS to an extent. The 2q31.1 region is resided mainly by HOXD genes. Previous studies in other cancer types have suggested a potential oncogenic role for HOXD genes, but the underlying mechanism is not clear [21,22,40]. Inhibition of the transcription of the HOXD locus was suggested to be relevant with tumor-cell immune escape [41]. Whether the HOXD genes contribute to tumor progression by shaping the immune microenvironment needs further investigation.

We also observed dysregulated immune response in the patients with relapse, which was marked by the upregulation of several immune-related pathways. A previous study deduced that immunosuppression was a critical risk factor for relapse [5]; however, the seemingly contradictory results in our study revealed that the broad immune level was not necessarily indicative of a poor prognosis, and the complexity of immune reactivity needed to be finely dissected to identify the essential determinant of relapse risk. Additionally, our samples were collected from primary tumors of stage I patients and, therefore, represented an early alteration in the TME, which revealed the vigorous combat between antitumor immunity and the tumor-intrinsic need for growth and survival. Moreover, we found that, although the overall level of immune response was high, Treg cells, which might shift the immune microenvironment to an inhibitory state, were enriched in PR. This controversial finding led to the assumption that these infiltrated Treg cells might not be able to exert inhibitory function to the immune response. Likewise, a previous study found the enrichment of Treg cells in ulcerative colitis, which was obviously characterized by vigorous inflammation, and suggested that these cells might represent a functionally impaired subgroup [26]. We also could not exclude the possibility that the Treg cells might reside in a naive state [42], considering that our samples were derived from early stages patients. However, lacking the single-cell-level resolution, we were not capable of further dissecting the heterogeneity of Treg cells to draw a more solid conclusion.

TATs represent a unique intermediate state between healthy and tumor tissues [43]. Robust predictive ability based on information extracted from TATs are proposed [44]. In our results, the TATs paired with NR and PR also presented with diversity in immune cell infiltration. We found that a defect of antigen processing and presentation in the TATs was prominent in PR_TATs. This impairment might circumvent the cytotoxicity of activated T cells and confer survival advantage to tumor cells which further acquire a chance of dissemination [45,46]. The underlying mechanism of immune deficiency, for instance, whether it was related to MSI or HLA-LOH, needs to be further investigated [47,48]. PR_TATs were also enriched for pathways of cell adhesion and junction. In fact, contradictory reports about the role of focal adhesion molecules in tumor invasion were documented [29,49]. By censoring the biological function of genes involved in the adhesion and junction, we also noticed multiples genes with oncogenic potential (www.gsea-msigdb.org, accessed on 1 December 2021). In summary, abnormality in TATs could also contribute to the cancer progression, and one should be cautious in using TATs as “normal” control.

While the genome of primary NSCLC has been well characterized, there has been considerably less of an analysis of recurrent specimens due to the challenge in acquiring samples. One of the strengths of our research is that our cohort used tumors after relapse to find out genomic variations and responsible pathways in RNA-seq. The recurrent lesions in our cohort manifested with inactive immune-related biological processes, therefore corroborating the immunosuppression state of recurrent lesions. In the analysis of immune cell infiltration, we found that monocytes were decreased in the RR lesions, and this might be ascribed to their weakened migration to the TME. As previously reported, inhibition of monocyte migration could promote the motility of tumor cells [50]. Moreover, our results indicated the transition of the immune process from a strong reaction at the early stage to a suppressive state at the advanced stage, thus suggesting that the application of immunological therapy might be different for early stage and advanced tumors.

The current therapy standards for NSCLC solely rely on the clinical staging. For example, adjuvant chemotherapy after complete surgical resection is not recommended for early stage NSCLC but might be considered as an option for stage IB patients according to the NCCN guidelines [51,52]. Whether or not to apply adjuvant therapy to NSCLC patients at stage I after surgical resection needed more reference beyond the TNM stage. A previous study reported that combining gene mutation with expression data could improve the outcome prediction of clinical outcomes [53]. Inspired by this, we established a model based on the comparison between NR and PR samples to select the predictive markers and calculate the risk score. Our results suggested that the transcriptional profile alone was enough to distinguish high-risk groups; therefore, deep phenotyping including multiple features might be a costly redundancy. Admittedly, further clinical trials are warranted to explore the adoption of adjuvant therapy in the high-risk patients.

## 5. Conclusions

Our study still suffered from several limitations. First, our cohort was very limited in the sample size, and this was a potential reason restricting the generalization of the model predicting relapse. Second, in the analysis of TATs, we did not define the precise distance of the regions sampled from the tumor or perform the comparison with truly normal tissues due to a shortage of samples from non-diseased individuals.

In summary, we characterized unique molecular signatures of stage I NSCLC with a high risk of recurrence, which is marked with by altered immune and metabolic environment, and revealed that the TATs of the primary lesions showed difference between patients with different relapse potential, suggesting that tumors might reshape the surrounding environment to facilitate their migration and re-seeding. We established a model which could effectively predict the risk of recurrence of stage I NSCLC after surgical resection in both the test and validation cohort. Further exploration in whether adjuvant therapy would improve the clinical outcome of stage I NSCLC patients with a high risk of relapse predicted by the model is required.

## Data Availability

The data in this research are available upon request.

## Supplementary Materials

The following are available online at https://www.mdpi.com/article/10.3390/cancers14133061/s1, Figure S1: Comparison of the prevalence of frequently mutated genes between PR and NR, Figure S2: Association between mutation status of USH2A and the DFS of stage I NSCLC from TCGA data, Figure S3: USH2A mutation is associated with TMB and TNB, Figure S4: Difference of TMB and TNB between NR and PR groups, Figure S5: Composition of single-nucleotide substitution signature, Figure S6: Difference of CNV score between NR and PR groups, Figure S7: KEGG pathway enrichment based on DEGs, Figure S8: Evaluation of immune cell infiltration, Figure S9: DEGs in PR_TATs compared with NR_TATs, Figure S10: Clonal evolution of paired PR and RR tumors, Figure S11: Pairwise comparisons of immune cell infiltration calculated by ssGSEA between PR and RR, Figure S12: Pairwise comparisons of immune cell infiltration calculated by CIBERSORT between PR and RR, Figure S13: Establishment of a prognostic model for predicting recurrence of stage I NSCLC, Table S1: Summary of clinical characteristics of patients, Table S2: Clinical information of tumor samples, Table S3: Clinical information of patients, Table S4: Quality-control data of WES, Table S5: Quality-control data of RNA-seq.

## Abbreviations

NSCLC	non-smallcell lung cancer
TME	tumor microenvironment
WES	Whole-exome sequencing
RNA-seq	RNA sequencing
TATs	tumor-adjacent tissues
OR	odds ratio
GSEA	gene set enrichment analysis
NES	normalized enrichment score
ssGSEA	single sample gene set enrichment analysis
DFS	disease-free survival
ROC	receiver operating characteristic
AUC	areas under the curve
PR	primary tumors with relapse
RR	recurrent tumors
NR	primary tumors without relapse
CNV	copy-number variation
TMB	tumor mutation burden
TNB	tumor neoantigen burden
DEGs	differentially expressed genes
KEGG	Kyoto Encyclopedia of Genes and Genomes
HR	hazard ratio
CI	confidence interval
