# Supplementary material for: Dysregulated Immune and Metabolic Microenvironment Is Associated with the Post-Operative Relapse in Stage I Non-Small Cell Lung Cancer
Source: Cancers (Basel). 2022 Jun 22;14(13):3061. doi: 10.3390/cancers14133061 (PMC9265031; doi:10.3390/cancers14133061)
Supplement: Supplementary file 1 [file cancers-14-03061-s001.zip › cancers-1728676 supplementary.pdf]

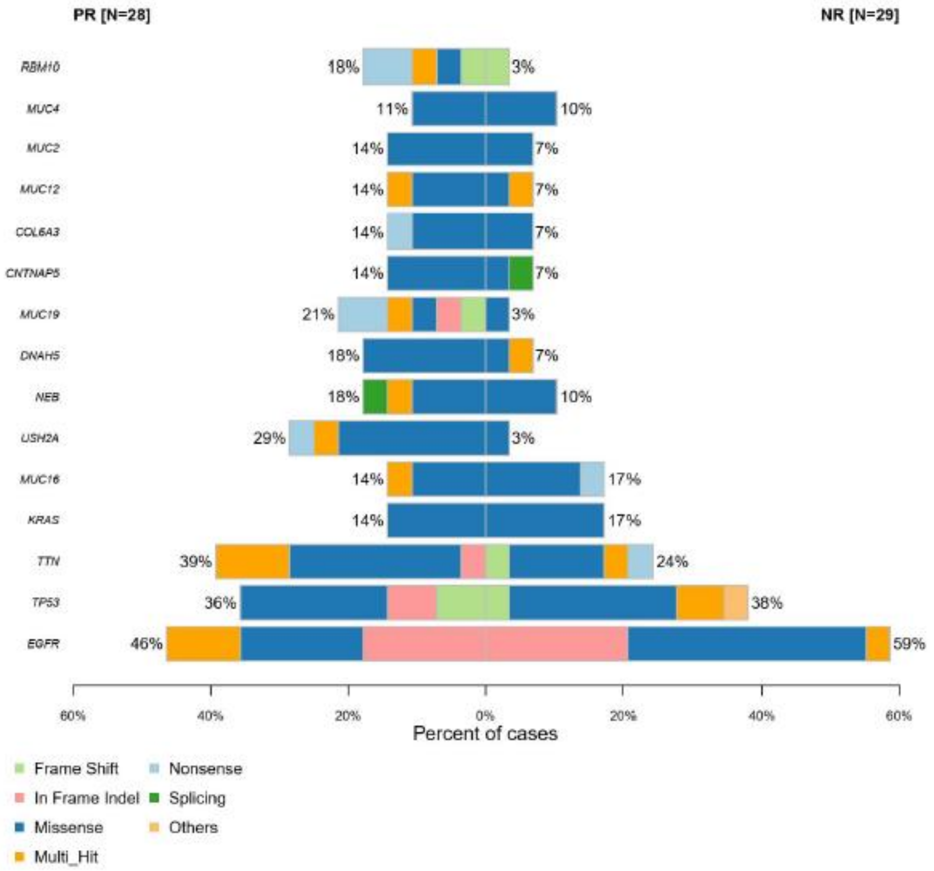

Figure S1. Comparison of the prevalence of frequently mutated genes between PR and NR.

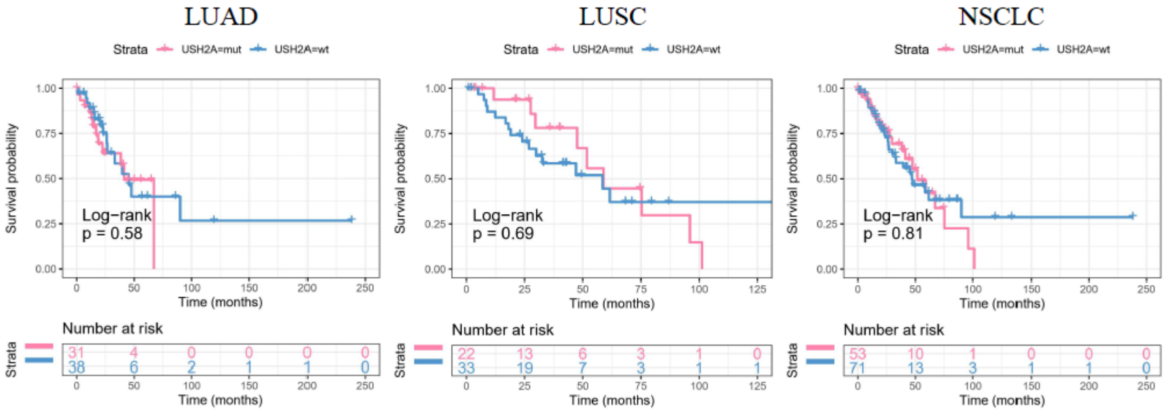

Figure S2. Association between mutation status of *USH2A* and the DFS of stage I NSCLC from TCGA data. Kaplan-Meier plot and Log-rank test showing no significant difference of DFS between USH2A mutant group and USH2A wildtype group in stage I LUAD (left), LUSC (middle) and NSCLC (right) cohorts respectively.

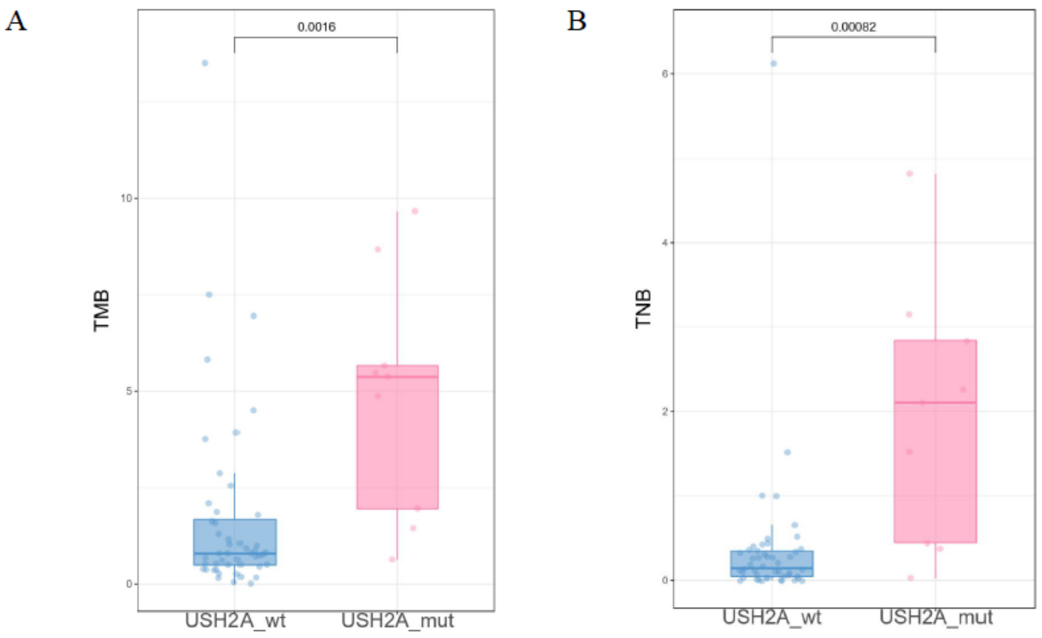

Figure S3. *USH2A* mutation is associated with TMB and TNB. (**A**) Boxplot showing the difference of tumor mutation burden between *USH2A* wildtype and *USH2A* mutant groups. (**B**) Boxplot showing the difference of tumor neoantigen burden between *USH2A* wildtype and *USH2A* mutant groups. The significance of difference was evaluated by Wilcoxon Rank-sum test.

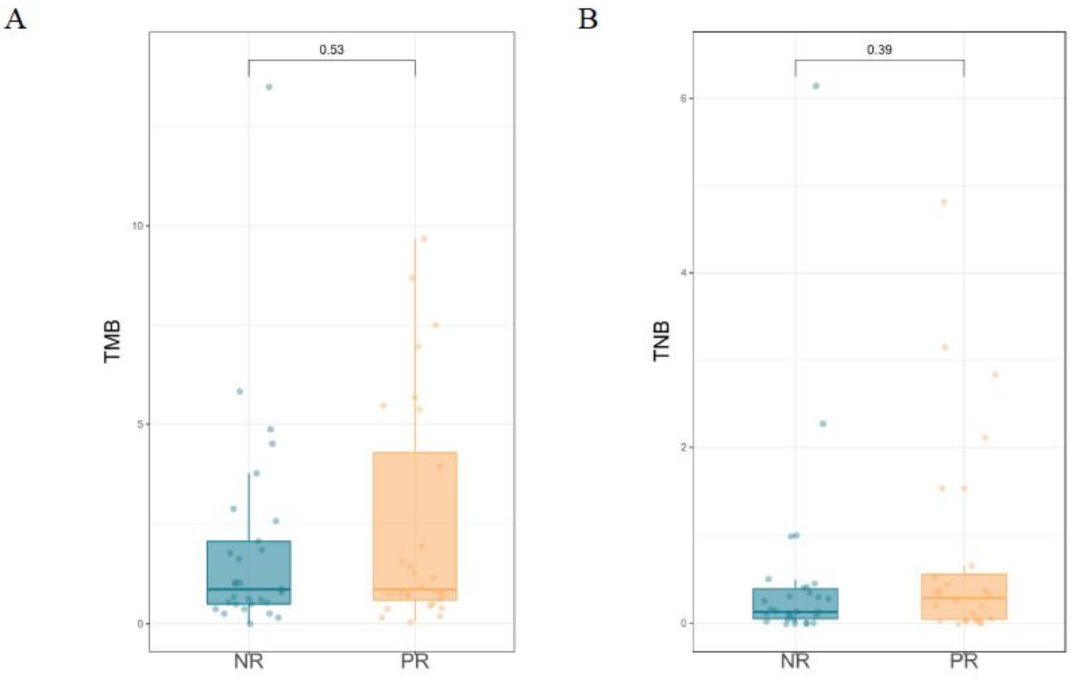

Figure S4. Difference of TMB and TNB between NR and PR groups. (**A**) Boxplot showing the difference of tumor mutation burden between NR and PR groups. (**B**) Boxplot showing the difference of tumor neoantigen burden between NR and PR groups. The significance of difference was evaluated by Wilcoxon Rank-sum test.

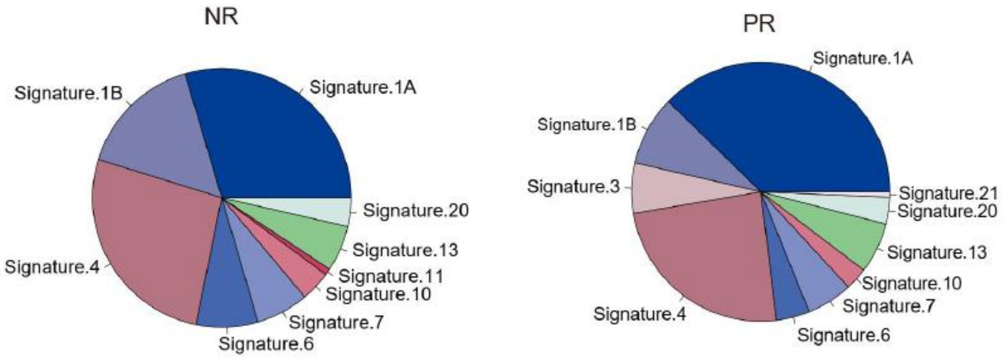

Figure S5. Composition of single-nucleotide substitution signature. Pie charts showing the composition of SBS signature in NR and PR respectively.

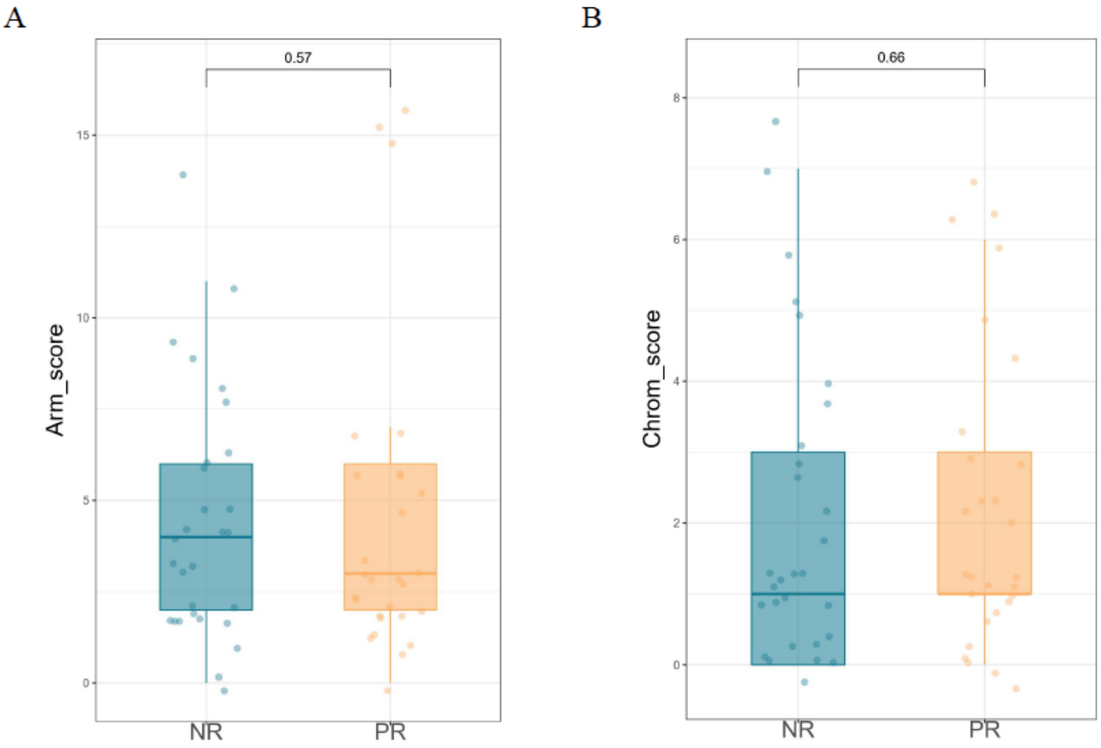

Figure S6. Difference of CNV score between NR and PR groups. (**A**) Boxplot showing the difference of Arm_score between NR and PR groups. (**B**) Boxplot showing the difference of Chrom_score between NR and PR groups. The significance of difference was evaluated by Wilcoxon rank-sum test. Arm_score and Chrom_score represent arm level and chromosome level CNV respectively. See methods for details.

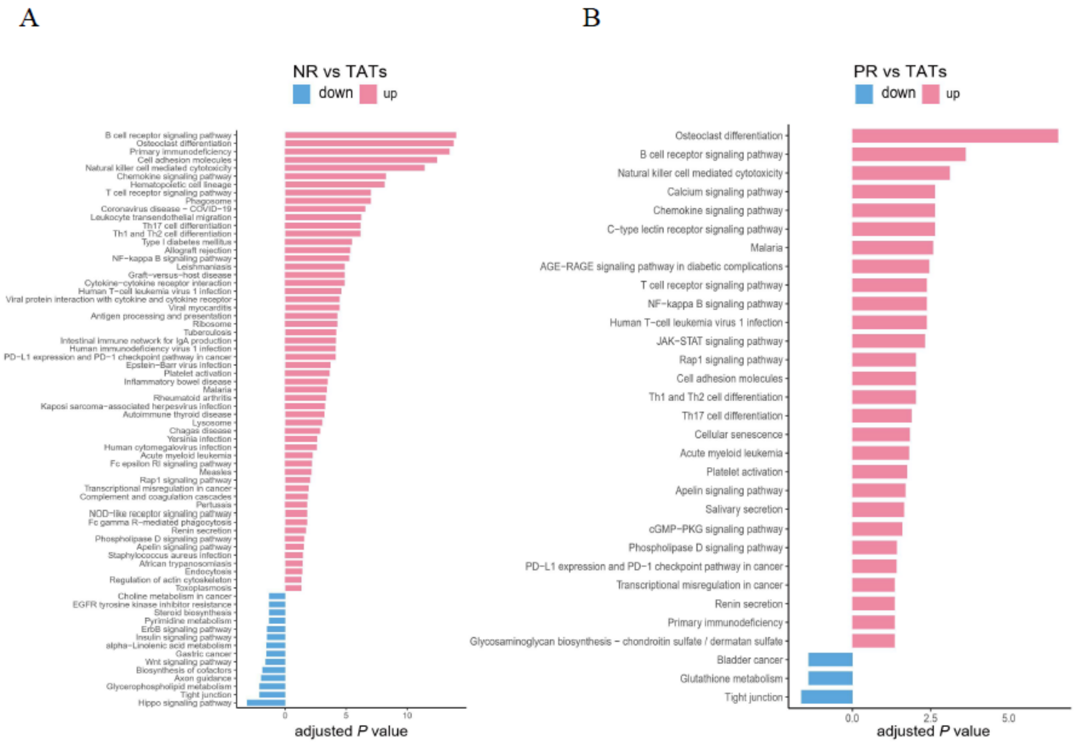

Figure S7. KEGG pathway enrichment based on DEGs. (**A**) KEGG pathway enrichment using down-regulated and up-regulated DEGs in NR tumors versus paired TATs. (**B**) KEGG pathway enrichment using down-regulated and up-regulated DEGs in PR tumors versus paired TATs.

A

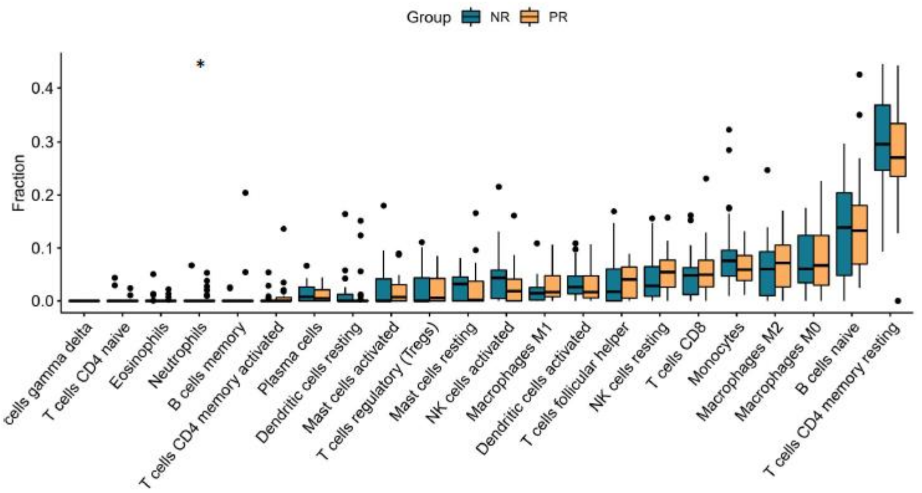

B

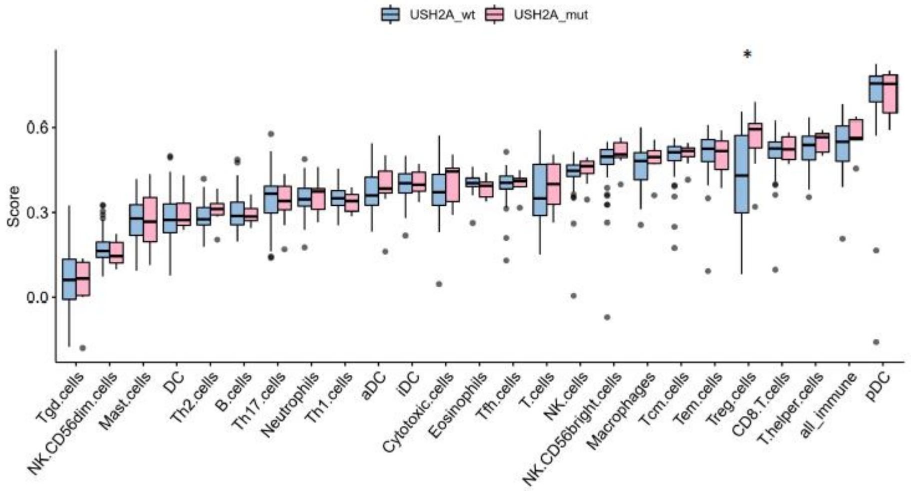

C

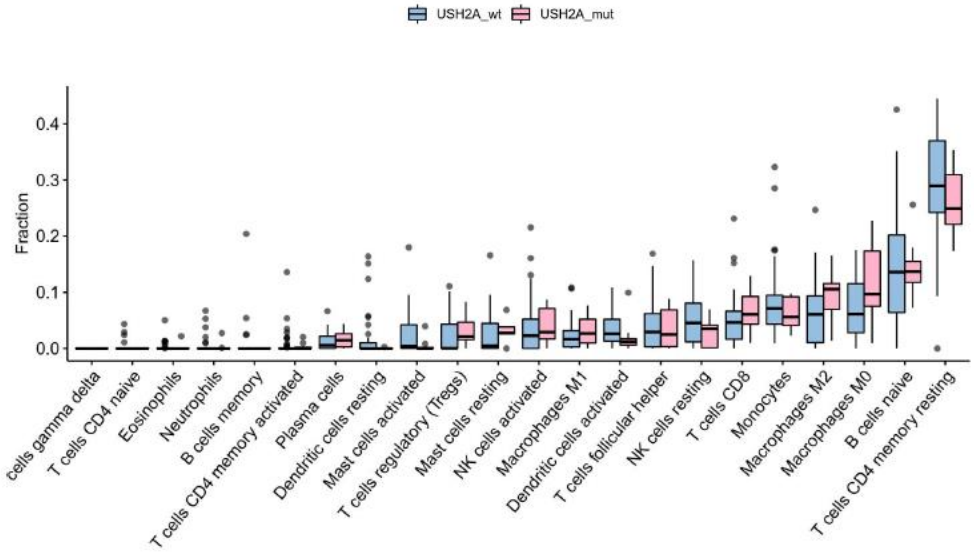

Figure S8. Evaluation of immune cell infiltration. (**A**) Boxplots showing the difference of immune cell infiltration between NR and PR evaluated by CIBERSORT. (**B**,**C**) Boxplots showing the difference of immune cell infiltration between USH2A wild type and USH2A mutant groups evaluated by ssGSEA (B) and CIBERSORT (C) respectively.

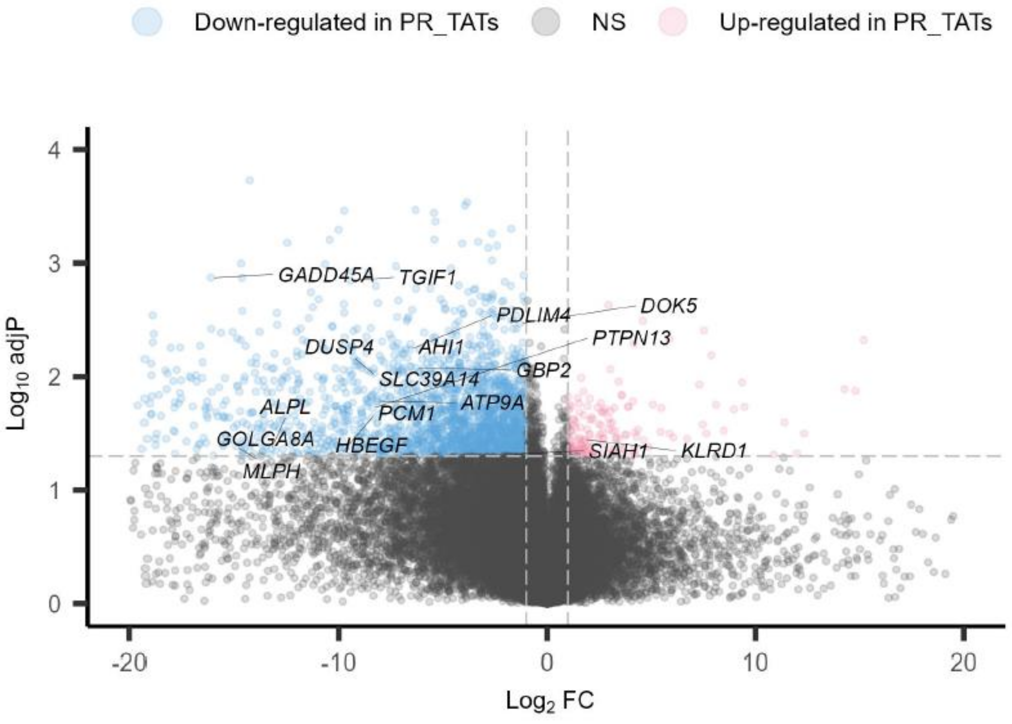

Figure S9. DEGs in PR_TATs compared with NR_TATs. Volcano plot showing the up-regulated (red) and down-regulated genes (blue) in PR_TATs compared with NR_TATs. Vertical and horizontal dashed lines represent the cutoff for Log2FC (1 for up-regulated and -1 for down-regulated) and adjusted P value (0.05). Genes as markers of immune cells (Figure 3B and C) are annotated.

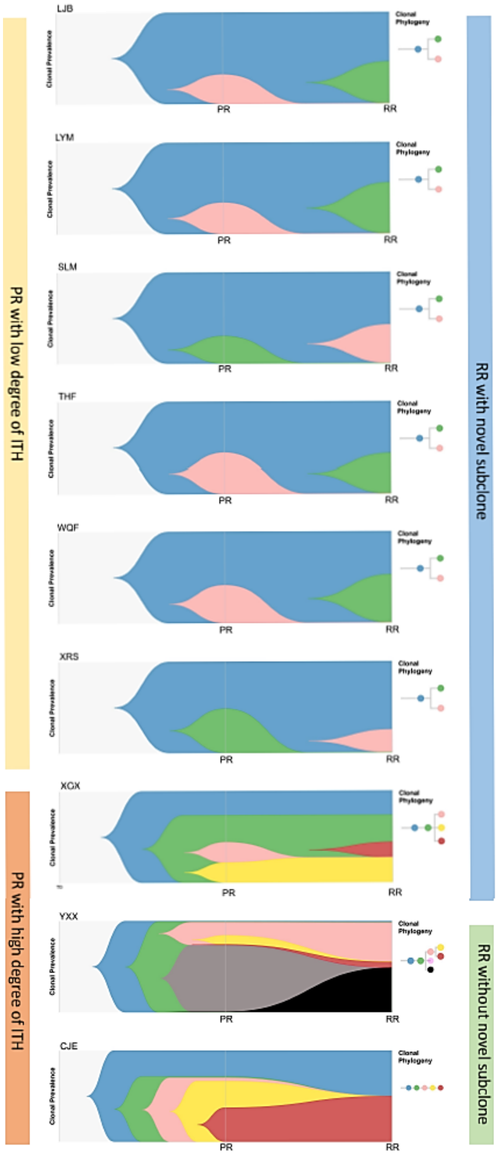

Figure S10. Clonal evolution of paired PR and RR tumors. Inference of subclones and the their phylogenetic structure in each sample are visualized. Subclones were inferred by PyClone. Phylogeny of subclones was constructed by CITUP. Visualization was realized by Timescape.

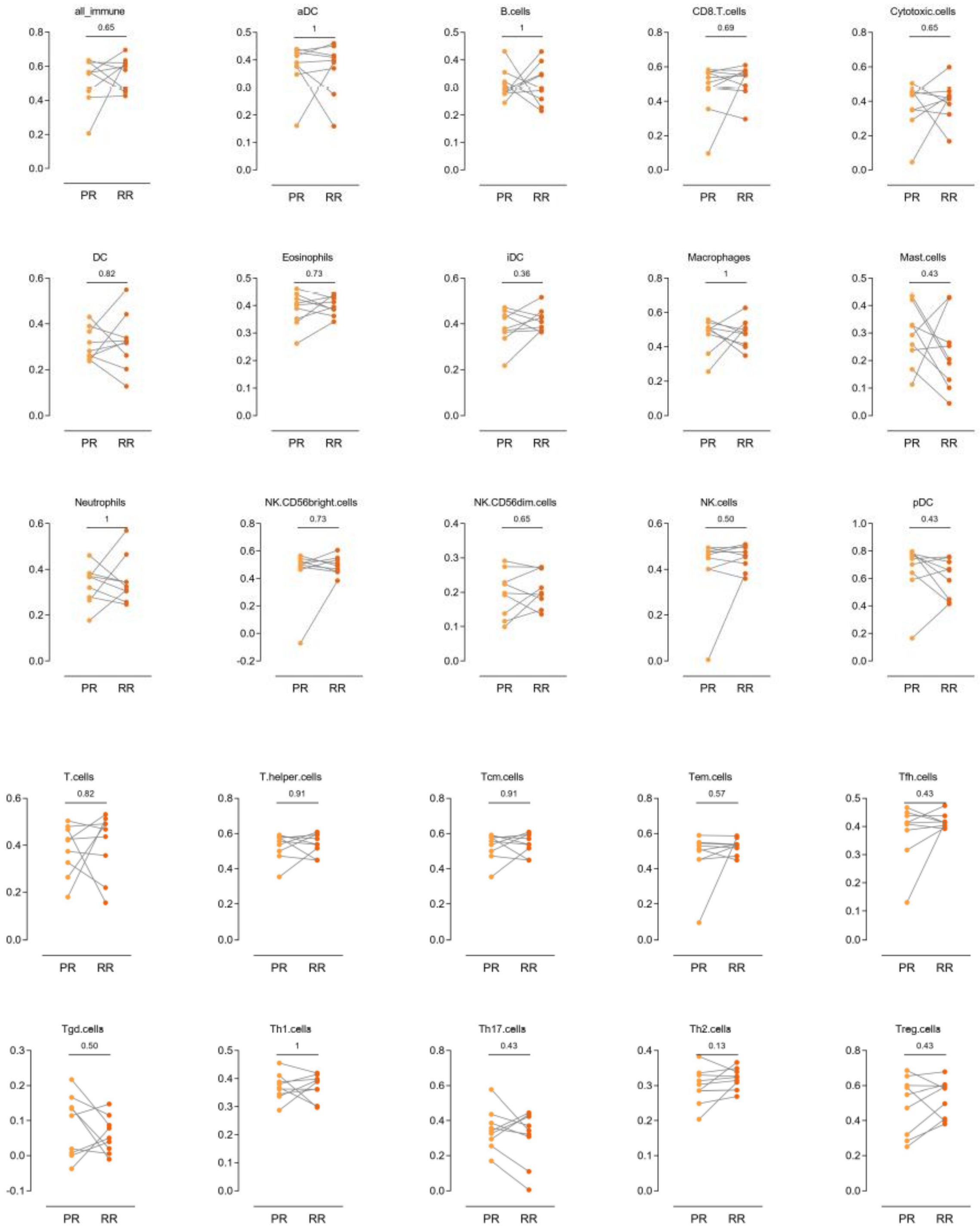

Figure S11. Pairwise comparisons of immune cell infiltration calculated by ssGSEA between PR and RR. The difference was evaluated by Wilcoxon matched-pairs signed rank test.

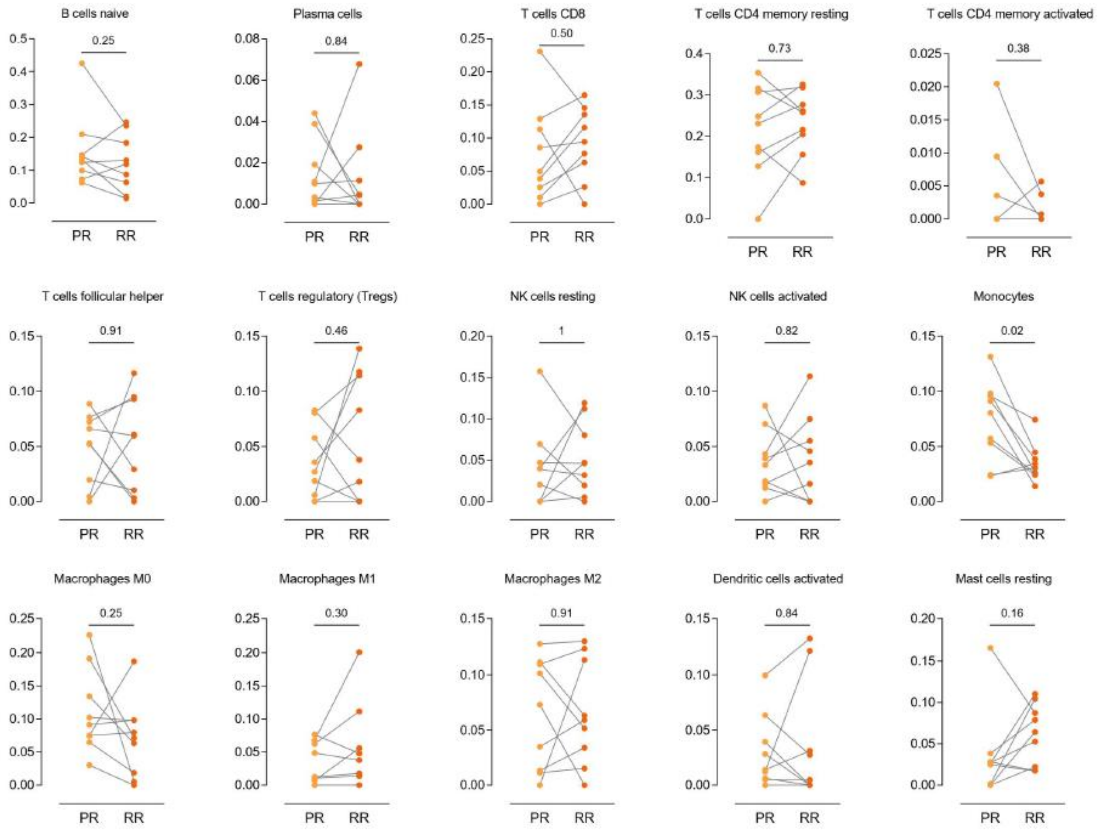

Figure S12. Pairwise comparisons of immune cell infiltration calculated by CIBERSORT between PR and RR. The difference was evaluated by Wilcoxon matched-pairs signed rank test.

A

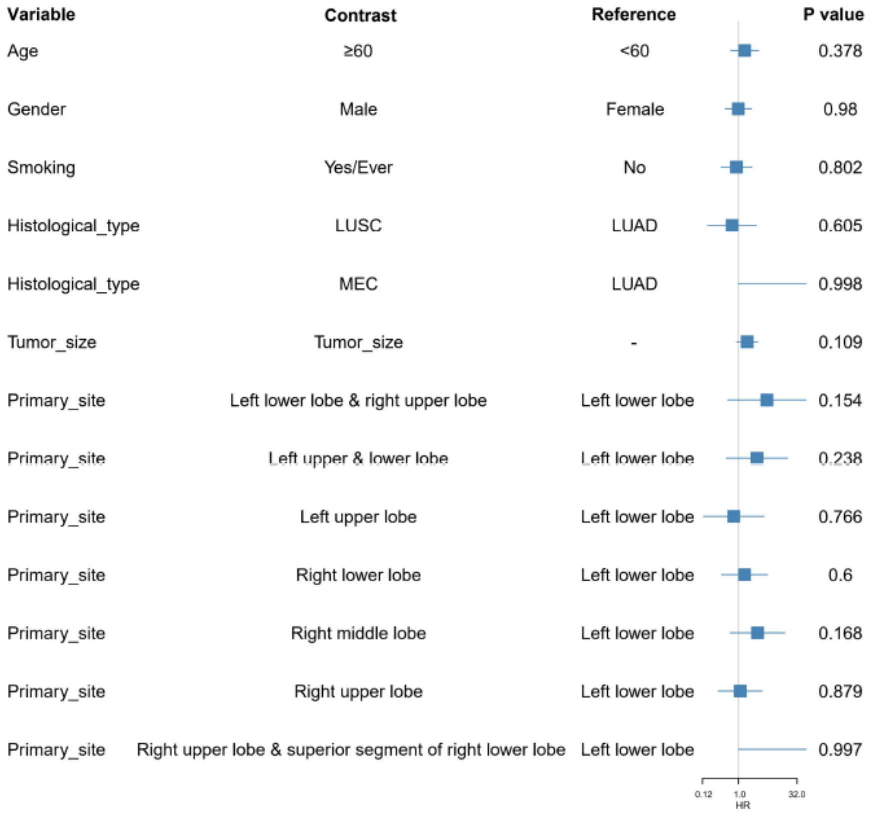

B

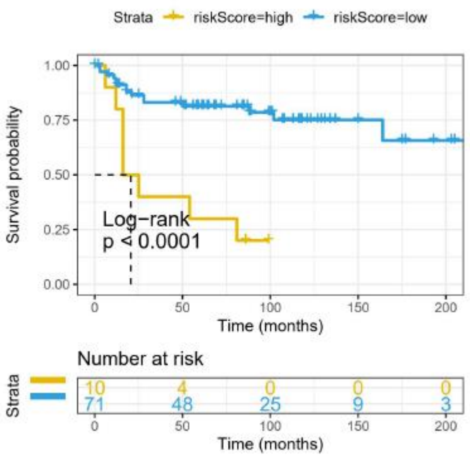

C

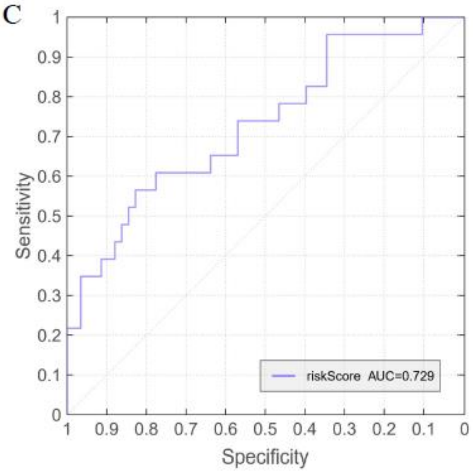

Figure S13. Establishment of a prognostic model for predicting recurrence of stage I NSCLC. (**A**) The influence of clinical characteristics on DFS was evaluated by univariate COX proportional hazards regression model. Forest plot indicates the HR value and the 95% confident interval. Variables such as MEC and Right upper lobe & superior segment of righ lower lobe show an infinite value of HR and therefore dissappear from the plot. (**B**) Kaplan-Meier curve showing the DFS between different risk groups of stage I LUAD from GSE30219. (**C**) ROC curve showing the recurrence prediction performance in stage I LUAD from GSE30219.
