# Supplementary material for: Dysregulated Immune and Metabolic Microenvironment Is Associated with the Post-Operative Relapse in Stage I Non-Small Cell Lung Cancer
Source: Cancers (Basel). 2022 Jun 22;14(13):3061. doi: 10.3390/cancers14133061 (PMC9265031; doi:10.3390/cancers14133061)
Supplement: Supplementary file 1 [file cancers-14-03061-s001.zip › cancers-1728676-supplementary/Table S1 clinical summary.pdf]

Dysregulated immune and metabolic microenvironment is associated with the post-operative relapse in stage I non-small cell lung cancer

Running title: Aberrant TME in stage I NSCLC cautions recurrence

Table S1 Summary of clinical characteristics of patients

**Characteristics**		**No relapse (N=29)**	**Relapse (N=28)**
Age	median range	61 (48-77)	61.5 (47-79)
Sex	Female	19 (65.5%)	18 (64.3%)
	Male	10 (34.5%)	10 (35.7%)
Smoking	No	22 (75.9%)	22 (78.6%)
	Yes/Ever	7 (24.1%)	6 (21.4%)
Stage	IA	3 (10.3%)	3 (10.7%)
	IB	18 (62.1%)	14 (50.0%)
	IC	8 (27.6%)	11 (39.3%)
Histology	LUAD	25 (86.2%)	26 (92.9%)
	LUSC	3 (10.3%)	2 (7.1%)
	MEC	1 (3.4%)	0 (0.0%)
